# mHealth Interventions to Support Prescription Opioid Tapering in Patients With Chronic Pain: Qualitative Study of Patients’ Perspectives

**DOI:** 10.2196/25969

**Published:** 2021-05-18

**Authors:** Michael Reece Magee, Amy Gray McNeilage, Nicholas Avery, Paul Glare, Claire Elizabeth Ashton-James

**Affiliations:** 1 Pain Management Research Institute Faculty of Medicine and Health The University of Sydney St Leonards Australia

**Keywords:** prescription opioids, chronic noncancer pain, tapering, digital health, support, mobile health technology, SMS, mobile phone

## Abstract

**Background:**

Patients with chronic pain who are tapering prescription opioids report a need for greater support for coping with symptoms of pain and withdrawal. Mobile health (mHealth) technologies (SMS text messaging– or app-based) have the potential to provide patients with educational, emotional, and motivational support for opioid tapering beyond what is offered by their health care provider. However, it is not known whether patients with chronic pain who are tapering opioids would be willing or able to engage with technology-based support.

**Objective:**

This study aims to examine patients’ use of mobile technologies in health care, interest in using mHealth support, preferences for the form and content of mHealth support, and potential barriers to and facilitators of engagement with mHealth support for opioid tapering.

**Methods:**

A total of 21 patients (11 women and 10 men; age range 29-83 years) with chronic noncancer pain on long-term opioid therapy who had recently initiated a voluntary opioid taper were recruited from primary and tertiary care clinics in metropolitan and regional Australia for a larger study of patients’ experiences of opioid tapering. Participants had been taking prescription opioids for a mean duration of 13 (SD 9.6; range 0.25-30) years at the time of the study. Survey items characterized participants’ typical mobile phone use and level of interest in mobile technology–based support for opioid tapering. Semistructured interviews further explored patients’ use of mobile technologies and their interest in, preferences for, and perspectives on potential barriers to and facilitators of engagement with mHealth support for opioid tapering. Two researchers collaborated to conduct a thematic analysis of the interview data.

**Results:**

All participants reported owning and using a mobile phone, and most (17/21, 81%) participants reported using mobile apps. The majority of participants expressed interest in SMS text messaging–based (17/21, 81%) and app-based (15/21, 71%) support for opioid tapering. Participants expected that messages delivering both informational and socioemotional support would be helpful. Participants expected that access to technology, mobile reception, internet connectivity, vision impairment, and low self-efficacy for using apps may be barriers to user engagement. Patients expected that continuity of care from their health care provider, flexible message *dosing*, responsivity, and familiarity with pain self-management strategies would increase user engagement.

**Conclusions:**

The results of this study indicate that patients with chronic noncancer pain may be willing to engage with SMS text messaging–based and app-based mHealth interventions to support opioid tapering. However, the feasibility and acceptability of these interventions may depend on how patients’ preferences for functionality, content, and design are addressed.

## Introduction

### Background

Chronic pain is one of the leading causes of disability worldwide, affecting approximately 1 in 5 adults [1-4]. Historically, chronic pain management has relied heavily on prescription opioid analgesics. However, the use of opioids in the treatment of chronic noncancer pain (CNCP) remains controversial. Evidence suggests that long-term opioid therapy has limited effectiveness in the management of CNCP [5,6] and carries significant risks, including physical dependence, tolerance (and associated dose escalation), and hyperalgesia (increased pain sensitivity) [7,8].

Current guidelines recommend that patients on long-term opioid therapy for CNCP should be gradually tapered off these medications under the guidance of a health care provider [5,6]. However, many patients are reluctant to change the way they manage their pain, particularly after long-term opioid use. Prescription opioid tapering is notoriously difficult, but it may be particularly difficult for patients with CNCP [9-11]. After prolonged use, even gradual reductions in opioid dosage may lead to withdrawal symptoms, including disrupted appetite, sleep, and mood (eg, agitation, anxiety, and restlessness); abdominal dysfunction; nausea; and muscle aches [11-13]. In addition to these withdrawal symptoms, patients with CNCP may also experience a temporary *flare-up* of their pain condition [12]. Faced with the dual challenges of coping with pain and withdrawal symptoms, it is not surprising that many patients with CNCP find it difficult to maintain motivation to continue opioid tapering [7,9-11].

Emerging data suggest that when patients volunteer to taper, a patient-centered, multimodal approach involving pain education, nonpharmacological pain self-management strategies, routine follow-up with a trusted health care provider, and support for coping with pain and withdrawal symptoms may improve outcomes [9,14-18]. However, patients face a number of obstacles in accessing comprehensive opioid tapering support. In addition to cost issues, multidisciplinary pain services are limited, and primary care providers often lack confidence in delivering nonpharmacological pain management advice. Furthermore, patients may be reluctant to admit their need for support with opioid tapering for fear of being stigmatized [6,8,10-12,14,16,19].

Mobile health (mHealth) interventions may be an effective means of overcoming many of these barriers to opioid tapering in patients with CNCP [20]. mHealth interventions have been found to be effective in supporting people to manage a variety of complex chronic health conditions, including diabetes, cardiovascular disease, and obesity [21-25]. mHealth has also been used effectively to support the cessation of addictive substances [26-28], to improve people’s ability to cope with chronic pain, and to enhance perceived quality of life [29-32]. One study found that a narrative (patient testimony) educational video about opioid tapering increased patients’ tapering self-efficacy to a greater degree than a pamphlet with the same information [33]. This study demonstrates how multimedia and digital platforms can play a role in increasing patients’ confidence and motivation to taper opioid medications.

The potential benefit of SMS text messaging–based interventions to support opioid tapering in patients with CNCP is supported by studies investigating the use of telephone-delivered messages to support chronic pain management, on the one hand, and opioid tapering, on the other. First, a 2010 study investigated the effect of delivering automated telephone voice messages to patients to remind them to use pain self-management skills after they completed an 11-week group pain management program. The researchers found that those who received the messages used fewer opioid medications 8 months after the pain management program compared with participants who did not receive digital aftercare [34]. Second, a 2020 study investigated the impact of receiving SMS text messaging–delivered cognitive behavioral pain self-management strategies on pain intensity and opioid use after surgery. Participants who received the SMS text messaging–delivered support reported lower pain intensity 2 weeks after surgery compared with a control group who received treatment as usual [35].

### Objective

Although these studies point to the potential of mHealth interventions to support prescription opioid tapering in individuals with chronic pain, there is currently no direct evidence that patients with chronic pain would be willing to engage with mHealth interventions as a means of support for opioid tapering and, if so, whether or not they would be able to engage with these technologies. This study aims to explore patients’ access to and use of mobile technology, interest in mHealth support for opioid tapering, preferences for the form and content of mHealth support, and potential barriers to and facilitators of engagement with mHealth support for opioid tapering. Using a combination of survey and interview methods to achieve these objectives, we also aim to elicit consumer perspectives to inform the design, content, and functionality of an mHealth intervention.

## Methods

### Setting

Participants were recruited from a private primary care practice in regional New South Wales and a public tertiary pain clinic in a metropolitan area in Australia between September 2019 and March 2020. The Human Research Ethics Committee of the Northern Sydney Local Health District approved the study (study 2019/STE00599).

### Inclusion and Exclusion Criteria

Eligible participants were those who were aged >18 years, were living with CNCP (pain persisting for ≥6 months), had been taking opioid medications for chronic pain for >3 months, and voluntarily commenced or planned to commence opioid tapering. Exclusion criteria included a history of opioid use disorder, major psychiatric conditions, insufficient English proficiency, or inability to provide informed consent. Patients with comorbid opioid use disorder were excluded from the study because the challenges faced by these patients when tapering are potentially more complex, requiring greater medical management, and the needs of this group are not necessarily shared by most patients tapering opioids for chronic pain [36].

### Design and Procedure

Data for this study were collected as part of a larger investigation into the experience of tapering in patients with CNCP. Patients who met the inclusion criteria were asked if they would be interested in hearing about the study. A researcher (AGM) contacted interested patients to obtain their informed consent to participate. Participants completed a very brief survey before or within the first few weeks of voluntarily tapering prescription opioids. Participants were later (during the first three months of their taper) interviewed (by AGM) and asked to elaborate upon their survey responses. A total of 2 independent researchers (MRM and CEAJ) analyzed the survey and interview data.

### Survey

The survey was designed to gather descriptive data on the sample of participants’ use of mobile phone technology (specifically SMS text messaging, apps, and health apps) and level of interest in engaging with mHealth support for opioid tapering (Multimedia Appendix 1). Specifically, participants were asked to respond to 2 key questions using a 5-point scale (1=no interest at all and 5=very interested): “How interested are you in receiving text messages with informative and supportive content while reducing your opioid medication?” and “How interested are you in using an app designed to support you in reducing opioid medication?” The survey also recorded demographic and clinical data, including age, gender, education, relationship status, duration of pain, and duration of opioid use.

### Interview

Semistructured interview questions were designed to further explore patterns of mobile phone and app use among participants in this cohort (eg, “How often do you use your phone?” and “At what times of day do you typically use your phone?”), identify reasons why participants expressed an interest or lack of interest in mHealth support for opioid tapering (eg, “You’ve said you would prefer SMS-based support over app-based support. Why is that?”), elicit participants’ preferences for the form and content of an mHealth intervention (“Do you have any thoughts on what kind of information would be helpful?” and “What advice would be helpful for someone who was about to taper their opioid medication?”), and explore potential barriers to and facilitators of engagement with mHealth support for opioid tapering (eg, “Why do you feel it would not be helpful for you?” and “Is there a time of day you might be more or less likely to look at it?”).

An interview guide was prepared as a prompt for interviewers to focus their discussion on issues pertinent to the aims of the study (Multimedia Appendix 1). However, interviewers were trained to use open-ended questions in response to participants’ answers to the stem questions to encourage elaboration (eg, “Tell me more about why you think you would not be interested in this sort of app?”). Data saturation was reached when participants no longer had additional perspectives to share when prompted on the semistructured interview items or open-ended follow-up questions.

### Participants

A total of 21 participants were recruited for this study. Participants were recruited until sample heterogeneity was achieved in terms of age, tapering experience, gender, education level, employment status, health care setting, pain duration, and opioid use duration. One participant withdrew from the study after completing the survey but before being interviewed for this study (discontinued tapering). Overall, 62% (13/21) of participants were recruited from a tertiary pain clinic, and 38% (8/21) of participants were recruited from primary care. Table 1 provides the demographic and clinical characteristics of the sample, including age, education, relationship status, employment status, pain duration, opioid medication duration, and oral morphine equivalent daily dose before tapering.

Most participants (17/21, 81%) reported prior experience with opioid tapering. Tapering experience was variable, including tapering from a very high dose to a moderate dose, with subsequent tapering to further reduce the dose. Others had successfully reduced or discontinued opioid medications in the past but had resumed opioid use after an accident or injury and were motivated to taper again. Participants were not recruited for this study based on their mHealth use or level of comfort with digital technology.

**Table 1:** Participant characteristics (N=21).

Characteristics			Values
Age (years), mean (SD; range)			55 (12.26; 29-83)
**Gender, n (%)**			
	Female	11 (52)	
	Male	10 (48)	
**Recruitment setting, n (%)**			
	Tertiary pain clinic	13 (62)	
	Primary care	8 (38)	
**Education, n (%)**			
	High school graduate	3 (14)	
	Vocational training	6 (29)	
	Bachelor’s degree	7 (33)	
	Postgraduate degree	2 (10)	
	Data missing or not reported	3 (14)	
**Relationship status, n (%)**			
	Single	2 (10)	
	Married	10 (48)	
	In a relationship	3 (14)	
	Widowed	2 (10)	
	Data missing or not reported	4 (19)	
**Employment status, n (%)**			
	Employed	4 (19)	
	Unemployed or not working	16 (78)	
	Data missing or not reported	1 (5)	
Pain duration (years), mean (SD; range)			13 (9.6; 0.25-30)
Duration of opioid treatment (years), mean (SD; range)			9.3, (7.5; 0.25-30)
Oral morphine equivalent daily dose (mg), mean (SD; range)			150 (229.29; 20-1080)

### Data Analytic Technique

Interview data were audio recorded and then transcribed verbatim into NVivo 12 (QSR International), a software platform for the qualitative coding and analysis of data [37]. A total of 2 researchers (MRM and CEAJ) conducted a mixed inductive-deductive thematic analysis. Thematic analysis was chosen as it provides a stepwise procedure for identifying the key features of a qualitative data set and is an appropriate methodology when there are clear research questions [37]. Furthermore, the data were considered to be generally of a manifest nature, in that the literal interpretations of the responses were deemed to accurately reflect the participants’ perspectives. Interview excerpts were coded, and initial themes were identified. The researchers independently completed conceptual mapping and thematic grouping of the data and collaboratively identified emergent higher-order themes and subthemes. The findings were discussed with a wider research group, in particular, as other members of the team had conducted the interviews and confirmed that accurate interpretation of results was imperative. Participants were not asked to review the results, as interview data were not of a complex phenomenological nature, and as such, it was considered that participant responses were reasonably clear and straightforward to interpret.

## Results

### Survey Results

All participants (21/21, 100%) reported using a mobile phone. Most participants (17/21, 81%) reported using mobile apps, including health and wellness apps (10/21, 47%). Overall, 81% (17/21) of participants reported being *interested* or *very interested* in SMS text messaging support for opioid tapering, and 71% (15/21) reported being *interested* or *very interested* in app support for opioid tapering (Table 2).

**Table 2:** Survey results.

Survey item and response option			Frequency, n (%)
**Use a mobile phone**			
	Yes	21 (100)	
	No	0 (0)	
**Interest in SMS text messaging support for opioid tapering**			
	Not interested at all	0 (0)	
	Not interested	3 (14)	
	Neither interested nor disinterested	1 (5)	
	Interested	16 (76)	
	Very interested	1 (5)	
**Use of mobile apps**			
	Yes	17 (81)	
	No	4 (19)	
**Use of health and wellness apps**			
	Yes	10 (48)	
	No	9 (43)	
**Interest in app support for opioid tapering**			
	Not interested at all	2 (9)	
	Not interested	2 (9)	
	Neither interested nor disinterested	0 (0)	
	Interested	13 (62)	
	Very interested	2 (9)	
	Missing responses	2 (9)	

### Interview Results

#### Patterns of Mobile Phone Use

During interviews, participants revealed that they most commonly used their mobile phones to communicate with others and did so via phone calls, SMS text messaging, and apps (eg, WhatsApp and Facebook Messenger). Participants reported using apps, including internet browsers, calendars, web-based booking platforms, games, banking, news, and social media. One participant reported using apps to record and listen to sessions with a psychologist for pain management support.

The frequency of phone use varied. Participants indicated that they had access to their phones all the time (“They’re kind of attached to you,” P18, male, 41 years, pain clinic, regional area), even if they did not always use them (“It’s just for emergencies,” P13, male, 51 years, pain clinic, metropolitan area)*.* Some participants reported using their phones mostly in the morning, whereas others reported more frequent phone and app use in the afternoons and evenings. One participant attributed reduced phone use in the morning to pain- and medication-related issues:

I struggle to get out of bed in the mornings because I don’t sleep well and that’s generally the point of time where the medication has worn off from the night before.

P18, male, 41 years, pain clinic, regional area

#### Interest in mHealth Support for Tapering

The proposed delivery of support via SMS text messaging or an app was perceived generally as positive:

I think just getting the support no matter what form it is in is helpful.

P08, female, 32 years, pain clinic, metropolitan area

Some participants reported that electronic delivery of motivation and encouragement would be welcome:

I think any form of motivation is helpful.

P10, male, 64 years, pain clinic, metropolitan area

A primary benefit of an mHealth intervention reported by participants was increased access to support for opioid tapering during periods when other services were not available (ie, out of office hours):

That’s where messages are going to work well...these [worries] can [come] at two in the morning and you need a response...It can be an automated thing.

P01, male, 60 years, pain clinic, regional area

One participant reported that an mHealth intervention would be beneficial as she had “nobody checking in to see how I am” over the Christmas period:

I think the mobile system would be good for that because it feels like you have some way of reaching someone or some way of someone reaching you to check in.

P08, female, 32 years, pain clinic, metropolitan area

Although participants were, in general, interested in using SMS text messaging–based or app-based support for opioid tapering, they also expressed a strong preference for clinician-delivered support either face-to-face or over the phone:

I like being able to I guess just talk...I tend to kind of ignore my texts sometimes if I’m busy.

P12, male, 46 years, pain clinic, metropolitan area

No I think it’s better to talk to somebody...No, I’d sooner talk over the phone.

P11, female, 83 years, primary care, regional area

I think the phone call thing is better than any social media, better than anything you just type in or anything because it’s human.

P04, male, 57 years, pain clinic, metropolitan area

There were concerns about how this would be interpreted if it was clear that the support was computer delivered:

If it’s just a text message, I’m not dumb, I know it’s just a computer sending it. If it gets sent out the same time every day, it’s a computer sending it.

P06, female, 59 years, pain clinic, metropolitan area

Participants reported some skepticism that automated messages would feel genuine:

If I think I’m getting just some random “great you’re doing well” and I know it’s been automatically generated, I probably wouldn’t be that tickled by it.

P02, female, 61 years, pain clinic, metropolitan area

Participants who did not express an interest in an mHealth intervention to support opioid tapering reasoned either that they had a strong desire for face-to-face support (“I think personally you need to be human to human,” P21, male, 53, primary care, regional area) or a preference for alternative sources of information and support:

I would probably prefer just reading information or just talking to someone before using an app. If there was a web page, I’d probably be likely to use the web page more.

P02, female, 61 years, pain clinic, regional area

However, one participant reported a preference for SMS text messaging rather than a phone call or face-to-face check in:

The text messaging I think would be really good because the thing that probably scares me most is to speak to someone about it.

P08, female, 32 years, pain clinic, metropolitan area

#### Preferences for mHealth Design and Functionality

In line with the preference for face-to-face and nonautomated services outlined earlier, several participants recommended that an option for personal follow-up be built into the service:

I think the mobile system would be good for that because it feels like you have some way of reaching someone or some way of someone reaching you to check in.

P08, female, 32 years, pain clinic, metropolitan area

Most participants did not express a strong preference for either SMS text messaging–based or app-based support. Participants who expressed a preference for SMS text messaging–based support indicated a higher level of familiarity with text messaging:

I can use [SMS] easier...because I don’t use my phone much for that sort of thing [apps].

P09, male, 61 years, primary care, regional area

Participants who expressed a preference for app-based support reasoned that apps may provide access to more content:

I’m guessing inside that app there would be probably more information than in the texts.

P18, male, 41 years, pain clinic, regional area

One participant stated that, although they were willing to read texts, they did not like to respond to them:

I just hate texting.

P16, female, 46 years, pain clinic, metropolitan area

#### Recommended Content of mHealth Support

Participants provided recommendations for content that they believed would be helpful in an mHealth intervention. Specifically, they expressed interest in receiving information about pain management during flare-ups and about withdrawal from opioid medications (informational support and reassurance) and messages of encouragement, motivation, and validation (socioemotional support and reassurance).

Several participants expressed a desire for information related to pain self-management strategies:

I think it would be really useful to have maybe different things on there like techniques to help. “If you’ve got really bad pain, why don’t you try this?”

P08, female, 32 years, pain clinic, metropolitan area

I think it would be nice to have like an app or texting or whatever that did embody like the mindfulness and the meditation and the feedback, so it’s all in one place.

P20, female, 63 years, pain clinic, metropolitan area

It’s about thought and relaxation...thought management.

P03, female, 29 years, pain clinic, regional area

One participant recommended tailoring the information to reflect the user’s stage of tapering to reassure them that what they were experiencing was normal:

Maybe “You’re on week 4 of your taper and you’re on this dose. Be mindful that you may experience this, this, and this.” So, give them a bit of warning. And “good ways to deal with those things are...” Then people know that they’re normal. Once people go “oh okay this is normal,” you take the fear out of it.

P08, female, 32 years, pain clinic, metropolitan area

Another participant agreed that knowing what to expect, particularly with regard to withdrawal symptoms, would be empowering:

I didn’t know what to expect. I thought it might just get worse and worse and worse for weeks. If I just had a time frame and it might be different...If I had been warned that you could get constipation and you’ll need to take precautions.

P02, female, 61 years, pain clinic, regional area

A number of participants commented more generally on the importance of social support when tapering, either from friends and family, a group within a treatment program, or clinicians. One participant recommended integrating electronic social support for patients who were tapering their opioid medication:

If you can push for a forum or something, I think that would be awesome.

P16, female, 46 years, pain clinic, metropolitan area

Participants’ views regarding the acceptability of mHealth interventions for opioid tapering were influenced by factors such as the frequency of the messages and pattern of delivery. One participant preferred receiving messages at night (“Probably early evening just as you’re winding down, to help me to wind down properly,” P04, male, 57 years, pain clinic, metropolitan area), whereas another was concerned about receiving too frequent or too repetitive messages. Their suggestion to counter this was for the digital service to be responsive to the needs of the user:

So maybe there is a text that’s in response to a word we send out. Like we need some affirmation...It could be geared to that more.

P01, male, 60 years, pain clinic, regional area

#### Potential Barriers to and Facilitators of mHealth Engagement

Several participants described perceived barriers toward phone-based digital interventions including limited phone reception or access to internet:

I don’t have Wi-Fi. I can’t afford it.

P06, female, 59 years, pain clinic, metropolitan area

I don’t think my old phone will support it.

P14, male, 56 years, primary care, regional area

Some participants spoke about vision impairment:

I use glasses and my phone is a very cheap phone. If it’s in the middle of the day I can’t read them.

P14, male, 56 years, primary care, regional area

For the older ones they can’t see stuff on their phone properly.

P16, female, 46 years, pain clinic, metropolitan area

Some participants reported that low confidence in using mobile devices and apps may be an obstacle to engagement with app-based interventions:

No, I wouldn’t know how [to use apps]...I’m not tech savvy.

P09, Male, 61 years, primary care, regional area

Relatedly, some participants suggested that ease of use and simplicity of content would facilitate engagement:

If it’s not too time consuming and it’s not complicated...your average person isn’t [so tech savvy] so it can’t be complicated.

P05, female, 58 years, pain clinic, metropolitan area

At the same time, one participant suggested that their confidence in using mHealth interventions could be improved if provided with technical support (P20, female, 63 years, pain clinic, metropolitan area).

Participants in the study had varying levels of familiarity and experience with pain self-management strategies, and one participant suggested that it may be difficult to engage with digitally delivered pain self-management advice if one is not already experienced in using them:

You don’t go driving on your own for the first time. You have to get the license. You need your lessons first...I would hate to have to do this [pain self-management] and not know how.

P01, male, 60 years, pain clinic, regional area

Participants predicted that their level of interest and engagement with mHealth support may fluctuate over the course of their taper depending on their mood, pain, and state of mind:

Whether I looked at it or not would depend I guess on my mood—how I’m feeling, where I am.

P18, male, 41 years, pain clinic, regional area

At the time you receive it you mightn’t be that open to it but in a few hours’ time when you’re starting to struggle a bit or the next day it may be something you open up again and read through.

P10, male, 64 years, pain clinic, metropolitan area

Whether the content was automated, personalized, and integrated with face-to-face treatment was identified as a potential barrier to engagement. Participants were concerned that receiving impersonal (automated and standardized) messages may not have the same impact as receiving individualized messages from a person in real time:

The text message, in reality that’s still a computer sending it, there’s not someone at the end of it that’s sending it. I don’t know whether it would just get irritating after a while because I know nothing is going to come of it, like there is no one at the other end. Or whether it would just jolt me out of feeling sorry for myself or something. I guess it depends how it’s worded.

P06, female, 59 years, pain clinic, metropolitan area

## Discussion

### Principal Findings

The results revealed that all participants owned and used mobile phones. Most participants reported using smartphone apps. The majority of participants expressed interest in SMS text messaging–based and app-based support for tapering prescription opioids. Mostly, participants held no strong preference for either SMS text messaging or app support. Variables potentially influencing acceptability included message simplicity, messaging frequency, time of delivery, responsivity to user needs, individual stage of tapering, and mental state There was a strong preference for clinician-delivered, individualized, real-time support over automated and standardized mHealth support. At the same time, however, a perceived benefit of mHealth was increased accessibility of opioid tapering support. Content recommendations included pain self-management and opioid withdrawal information as well as encouragement, motivation, and validation. Perceived barriers included limited phone or internet reception and low confidence in using mobile devices and apps. These results suggest that SMS text messaging–delivered or app-delivered support may be acceptable, feasible, and even helpful to individuals living with CNCP who are tapering opioids. However, participants identified a number of barriers to engagement that may be critical to address in the development of such an mHealth intervention.

### Implications for Design and Functionality

mHealth interventions are typically delivered via an app or SMS text messaging. Participants identified barriers such as access to technology, mobile reception, internet connectivity, vision impairment, and low confidence in using apps. Most of these obstacles can be overcome by using an SMS text messaging–based intervention rather than an app-based intervention. SMS text messaging–based interventions are very simple and easy to use and do not require an internet connection, and messages tend to be shorter and therefore less burdensome to read.

Participants suggested a number of factors that might influence engagement with mHealth interventions for prescription opioid tapering. In particular, participants expressed a desire to continue to receive care from their health care provider, pointing to the importance of ensuring that mHealth interventions are used as an adjunct to, rather than a replacement of, the patient-provider communication. Relatedly, some participants expressed concern that support received via text messages would feel impersonal or ingenuine. In response to this (common) concern, many mHealth interventions now personalize SMS text messaging content by, for example, addressing users by their preferred name or by tailoring messaging to the interests of the user (eg, “Hi Sam, doing things we enjoy helps us to feel good, so we feel less pain. Make plans to go for a hike, or perhaps meet a friend for coffee.”) These personalization strategies have been found to increase engagement with mHealth interventions [21,38].

Participants also indicated that their engagement with text messages may fluctuate and suggested that flexible message *dosing*—the ability to change the frequency of messages they receive depending on their mood, symptoms, coping, and need—may enhance the acceptability of an mHealth intervention for opioid tapering. It was also suggested that responsivity—the ability to request support *on demand*—would enhance the acceptability of an mHealth intervention. These concerns are not uncommon, and research suggests that user engagement can be enhanced by allowing users a degree of control over the frequency of messages they receive, either up front (initially) or after a standardized *loading* dose [21].

Another method of tailoring the frequency of messages to user demands is to allow users to text prompts to the program when they wish to receive a message. The content of *on-demand* messages can be determined by the user based on established keywords. For example, in response to texting *pain*, the user might receive a message offering pain coping strategies (eg, “breathe in slowly to a count of four, breathe out for six”) or informational reassurance (eg, “pain may flare up for many reasons—not necessarily due to tapering”). This functionality is common and feasible in mHealth technology [28,30].

### Implications for mHealth Intervention Content

Participants made 2 key recommendations for the content of an mHealth intervention to support people similar to them who were tapering opioids that were prescribed for CNCP. First, they recommended that users would benefit from messages about chronic pain management, opioid tapering, and strategies for coping with pain and withdrawal symptoms. This perspective concords with research demonstrating the positive association between giving patients informational or *cognitive* reassurance and pain management outcomes. The results of a systematic review found that patients who were provided with an explanation for their pain condition and reasons for their treatment advice experienced less distress and showed greater symptom improvement [39,40].

At the same time, however, participants also noted that it may be difficult to understand and engage with information about coping with pain and symptoms of withdrawal via text if one does not already have some familiarity with these concepts. This concept is in line with previous research that demonstrated reduced opioid medication use in a group of patients who received automated telephone voice messages following an 11-week group pain management program [34]. This highlights the value of mHealth reminders in using prelearned skills. This perspective also aligns with research demonstrating that people are more likely to remember messages that are familiar and messages that they agree with [41,42]. People tend to ignore information they do not understand or that conflicts with their existing beliefs and attitudes [43]. Hence, engagement with informational messages may require users to first have foundational pain and opioid tapering education covering concepts such as “what is pain?,” “how is pain managed?,” “reasons for tapering opioids,” and “what to expect.” Although there is some evidence that SMS text messaging–based mHealth support alone may reduce pain intensity and opioid use after surgery [35], it is likely that a pre-educational video would be of benefit [33].

Participants also suggested that messages offering social or emotional support may be beneficial and expressed a need for encouragement and validation. This is consistent with research demonstrating that validation—having one’s feelings and experience acknowledged without judgment—significantly reduces distress, elevates positive mood, and increases pain tolerance [44]. It is theorized that by reducing the levels of physiological arousal, validation sets the stage for behavior change [45]. Hence, the inclusion of messages of validation and support for emotional distress in the content of an mHealth intervention may indirectly help to facilitate behavior change.

### Limitations

A key limitation of this study is that participants reported their *expectations* of the feasibility and acceptability of mHealth interventions to support prescription opioid tapering rather than their actual *experiences* with an mHealth intervention. Likewise, the experience of tapering that an individual participant had, both their previous experiences of tapering and the stage of tapering when they were interviewed, may have influenced their responses regarding perceptions toward mHealth support. It is possible that participants’ expectations are not congruent with their experience [46], and future research will be needed to evaluate the experience of using mHealth to support prescription opioid tapering in people with CNCP. However, as a first step, the results of this study suggest that in our sample of people living with chronic pain who have been on long-term opioid therapy, there is an interest in piloting this mHealth intervention to support them in their efforts to taper prescription opioids.

We acknowledge that the interview guide contained prompt questions that were closed ended (eg, “Would you be interested in receiving SMS messages with informative and supportive content when you are reducing your opioid medication?”). Although interviewers were trained to follow up any closed-ended questions with an open-ended question, it is possible that closed-ended or leading questions may have biased patients’ responses.

This study was conducted within the context of a larger study, which determined the sample size of this study. Certainly, the results of our descriptive (quantitative) survey would be strengthened with further sampling. However, the goal of qualitative data collection was to canvas a variety of perspectives on mHealth support for opioid tapering rather than to evaluate the proportion of people living with chronic pain who share certain perspectives. For this purpose, our sample size was adequate.

This study was conducted before the COVID-19 pandemic in Australia. As a result of the pandemic and associated social distancing measures, Australians have become more familiar with digital health technologies (ie, telehealth). It is possible that in the interests of maintaining social distancing and leveraging some of the efficiencies of digitally delivered health care, Australians may be more willing to engage with digital health interventions now more than ever.

### Future Directions

The results of this study will be used to inform the development of a user-centered mHealth intervention to support people who are tapering prescription opioids for CNCP. Consistent with the existing guidelines for the development of mHealth interventions, the next stage of our research will involve co-designing and testing of intervention content (messages) in partnership with clinical experts as well as end users [38]. Table 3 summarizes the key findings regarding barriers to and facilitators of patients’ engagement and suggests implications of the design, content, and functionality of an mHealth intervention to support prescription opioid tapering.

**Table 3:** Summary of perceived barriers to and facilitators of patients’ engagement with implications for mobile health design, functionality, and content.

Theme			Implication		Recommendation
**Barriers to engagement**					
	Internet access	Functionality		SMS text messaging–based intervention may be more feasible than app-based intervention for this population	
	Access to smartphone technology	Functionality		SMS text messaging–based intervention may be more feasible than app-based intervention for this population	
	Digital literacy or confidence in using SMS text messaging versus apps	Design and functionality		SMS text messaging–based intervention may be more feasible than app-based intervention for this population	
	Vision impairment	Design		SMS text messaging–based intervention may be more feasible than app-based intervention for this population	
	Reservation about app use	Design		SMS text messaging–based intervention may be more feasible than app-based intervention for this population	
**Facilitator of engagement**					
	Maintaining contact with health care provider	Functionality		Communicate to users that the purpose of the intervention is to provide support in addition to that delivered by the health care provider	
	Fluctuating dose (support) needs	Design		User controls frequency of messaging after an initial *standardized* dose	
	Fluctuating content needs	Functionality		User can request support with specific issues by texting keywords to the server (eg, “crave,” “anxious,” or “pain”)	
	Familiarity with pain management strategies	Design		Standardized pre-education may be needed to ensure that patients have a basic understanding of pain management and reasons for opioid tapering before they receive supportive messages	
	Desire for individualized care	Functionality and content		Personalization of SMS text messaging content. Use of name and message tailored to demographic details of user	
	Predictability	Functionality		Variability in the time of day the messages are sent can increase attention and engagement	
	Socioemotional reassurance	Content		Validating message content that aims to normalize concerns that participants may have in their tapering (eg, “It’s natural to worry about pain increasing”)	
	Informational reassurance	Content		Informational message content to educate on nonopioid pain management (eg, “opioids are proven to relieve acute pain but not chronic pain”)	

### Conclusions

The results of this study indicate that patients with CNCP expressed interest in engaging with an mHealth intervention to support prescription opioid tapering. Interviews with this diverse group of potential users revealed barriers to feasibility and acceptability to be addressed and offered insights into factors that may increase engagement with this mHealth intervention. Future research will evaluate whether mHealth interventions improve patients’ confidence in their ability to manage their pain and maintain their quality of life while tapering opioid medications.

## Appendix

Multimedia Appendix 1 Survey and semistructured interview guide.

## Abbreviations

CNCP: chronic noncancer pain
mHealth: mobile health
