# Supplementary material for: mHealth Interventions to Support Prescription Opioid Tapering in Patients With Chronic Pain: Qualitative Study of Patients’ Perspectives
Source: JMIR Form Res. 2021 May 18;5(5):e25969. doi: 10.2196/25969 (PMC8170552; doi:10.2196/25969)
Supplement: Multimedia Appendix 1 [file formative_v5i5e25969_app1.docx]

# **Survey and Semi-Structured Interview Guide**

This is a Multimedia Appendix to a full manuscript published in the J Med Internet Res. For full copyright and citation information see http://dx.doi.org/10.2196/jmir.25969

**Survey of mobile phone use and interest in mHealth**
Instructions:

We are researching the use of mobile phone technology (e.g. SMS messages, apps) to support patients who are reducing their opioid medications. Your answers to these questions will help us to improve this research.

1. Do you use a mobile phone? Please mark one option.

O Yes O No

1. How interested are you in receiving SMS text messages with informative and supportive content when you are reducing your opioid medication? Please mark one option.

O Not interested at all

O Not interested

O Neither interested nor disinterested

O Interested

O Very interested

1. Do you use mobile phone applications (apps) on your phone? Please mark one option.

O Yes O No

1. Do you use health/wellness related apps? Please mark one option.

O Yes O No

1. How interested are you in using an app designed to support you in reducing opioid medication? Please mark one option.

O Not interested at all

O Not interested

O Neither interested nor disinterested

O Interested

O Very interested

##

## **Interview Guide**

### **Acceptability**

Would you be interested in receiving SMS text messages with informative and supportive content when you are reducing your opioid medication?

Do you think you would find this service helpful for reducing opioid medication?

Would you be interested in using a mobile phone application (app) designed to support you in reducing opioid medication?

Do you think you would find this app helpful for reducing opioid medication?

### **Feasibility**

Do you use a mobile phone?

How often do you use your phone?

What are the most common ways in which you use your mobile phone (e.g. work, social, family, appointments)?

What times of the days are you most active in using your phone?

Are there any times of the day in which you do not use your mobile phone?

Are there any times of the day in which you would prefer not to use your mobile phone?

Do you use SMS text messages?

Do you use any phone applications? If so, what are the main ones you use (e.g. Facebook, Instagram, Twitter, Google Calendar, Gmail, etc.)?

### **Prompts Regarding Content and Acceptability**

Was there anything that you know now that you wish you had known at the beginning of the taper?

What advice would you give to someone about to taper down their opioid medication?

Did you find my phone calls helpful (or not)?

Do you think text messages would be just as helpful/unhelpful?
